# Impact of clinical trial participation on survival in patients with castration-resistant prostate cancer: a multi-center analysis

**DOI:** 10.1186/s12885-018-4390-x

**Published:** 2018-04-26

**Authors:** Kyo Chul Koo, Jong Soo Lee, Jong Won Kim, Kyung Suk Han, Kwang Suk Lee, Do Kyung Kim, Yoon Soo Ha, Koon Ho Rha, Sung Joon Hong, Byung Ha Chung

**Affiliations:** 1 Department of Urology, Gangnam Severance Hospital, Yonsei University College of Medicine, 211 Eonju-ro, Gangnam-gu, Seoul, 135-720 Republic of Korea; 2 Department of Urology, Severance Hospital, Yonsei University College of Medicine, Seoul, Republic of Korea

**Keywords:** Clinical trial, Prostatic neoplasms, Castration-resistant, Survival

## Abstract

**Background:**

Clinical trial (CT) participation may confer access to new, potentially active agents before their general availability. This study aimed to investigate the potential survival benefit of participation in investigational CTs of novel hormonal, chemotherapeutic, and radiopharmaceutical agents in patients with castration-resistant prostate cancer (CRPC).

**Methods:**

This multi-center, retrospective analysis included 299 consecutive patients with newly diagnosed, non-metastatic or metastatic CRPC between September 2009 and March 2017. Of these, 65 (21.7%) patients participated in CTs pertaining to systemic treatment targeting CRPC and 234 (78.3%) patients received pre-established, standard systemic treatment outside of a CT setting. The survival advantage of CT participation regarding cancer-specific survival (CSS) was investigated.

**Results:**

An Eastern Cooperative Oncology Group performance status (ECOG PS) ≥2 at CRPC diagnosis was found in a lower proportion CT participants than in non-participants (4.6% vs. 14.9%; *p* = 0.033). During the median follow-up period of 16.0 months, CT participants exhibited significantly higher 2-year CSS survival rates (61.3% vs. 42.4%; *p* = 0.003) than did non-participants. Multivariate analysis identified prostate-specific antigen and alkaline phosphatase levels at CRPC onset, Gleason score ≥ 8, ECOG PS ≥2, less number of docetaxel cycles administered, and non-participation in CTs as independent predictors for a lower risk of CSS.

**Conclusions:**

Patients diagnosed with CRPC who participated in CTs exhibited longer CSS durations than non-participants who received pre-established, standard systemic therapy outside of a CT setting. Our findings imply that CT participation is associated with CSS, and that CT participation should be offered to patients with CRPC whenever indicated.

## Background

In line with advances in clinical research, the treatment of castration-resistant prostate cancer (CRPC) has evolved, with the development of novel hormonal, chemotherapeutic, radiopharmaceutical, and immunotherapeutic drugs [1]. Approval of these agents was based on the results of large, well-designed, randomized phase III clinical trials (CTs) that demonstrated improvement in overall survival [2–5]. However, there is still an unmet need to provide individualized therapeutic options, and the requirement for novel agents based on various pathways and targets continues to exist.

Participation in CTs may confer access to new, potentially active therapeutic agents before their general availability. Moreover, these investigational agents may be the best current treatment option for a subset of patients. Currently, there is ongoing research into and development of novel agents targeting CRPC, including androgen receptor inhibitors, cytochrome P450 17 inhibitors, targeted agents, vaccines, and poly-ADT-ribose polymerase inhibitors [6–8]. A randomized CT is a crucial step in the development of new cancer treatments, and as with previously approved drugs, the efficacy of these novel agents will need to be confirmed by adequate statistical power through CTs before their application in clinical practice.

Physicians offer enrollment in CTs assuming that survival benefits may be obtained from participation. Participating in CTs may also provide patients with hope of an individualized survival benefit conferred by the use of potentially effective agents that would not have been received outside the trial setting. Indeed, several studies have shown entry into cancer CTs to be associated with increased survival rates [9, 10]. On the other hand, concerns around the uncertainty associated with the experimental nature of CTs, the randomization process, unknown potential toxicity, and the time delay until proven standard therapy is available are documented barriers to enrollment [11–13].

With advances in understanding the mechanisms underlying castration-resistance and disease progression, CTs of investigational drugs targeted at CRPC will continue. Ideally, CTs should be offered as the best treatment option for patients based on evidence that participation may improve survival outcomes. Indeed, several investigations have reported a favorable overall trend with CT entry [14–16]. However, data on whether CTs targeted at CRPC may confer benefit in regard to survival are limited. The objective of this retrospective study was to determine the independent cancer-specific survival (CSS) advantage of participation in investigational CTs of hormonal, chemotherapeutic, and radiopharmaceutical agents targeted at CRPC.

## Methods

### Study population

A multicenter, retrospective analysis was performed using a prospectively collected database of 331 consecutive patients who were diagnosed with non-metastatic or metastatic CRPC between September 2009 and March 2017. Prostate cancer staging was based on the 7th American Joint Committee on Cancer TNM system, with the definition of bone metastasis based on either demonstrable metastatic deposits on imaging studies (bone scan, computed tomography, magnetic resonance imaging, or positron emission tomography) or by pathological confirmation. Patients were excluded from the analysis if they met any of the following criteria: incomplete clinical data (*n* = 14), lost to follow-up (*n* = 10), or unknown cause of death (*n* = 8).

Once diagnosed with CRPC, patients’ eligibility for participation in available CTs pertaining to novel hormonal, chemotherapeutic, and radiopharmaceutical investigational agents was assessed. The eligibility criteria for 18 CTs are listed in Table 1. Patients who did not meet the eligibility criteria or who refused to participate in CTs received systemic treatment according to standard U.S. Food and Drug Administration-approved dose and schedule. The choice and sequencing of standard agents were based on physician discretion and patient preference. Each agent was continued until the occurrence of radiographic disease progression, intolerable side-effects, patient refusal, or death. Serum prostate-specific antigen (PSA) measurements were performed every 1 to 3 months, and computed tomography and bone scans were performed every 2 to 4 months. This study was approved by the Yonsei University Health System Institutional Review Board after a review of the study protocol (2017–0186-001).

**Table 1:** Clinical trial protocols included in this analysis

*NCT identifier*	*Trial title*	*Phase*	*Reference*
NCT01946204	A Multicenter, Randomized, Double-Blind, Placebo-Controlled, Phase III Study of ARN-509 in Men With Non-Metastatic (M0) Castration-Resistant Prostate Cancer	Phase III	Apalutamide versus placebo
NCT00744497	A Randomized Double-Blind Phase 3 Trial Comparing Docetaxel Combined With Dasatinib to Docetaxel Combined With Placebo in Castration-Resistant Prostate Cancer	Phase III	Dasatinib, docetaxel, prednisone versus placebo, docetaxel, prednisone
NCT02057666	A Phase III, Randomised, Double-Blind, Placebo-Controlled Study Of Tasquinimod In Asian Chemo-Naïve Patients With Metastatic Castrate-Resistant Prostate Cancer	Phase III	Tasquinimod versus placebo
NCT01234311	A Phase 3 Randomized, Double-Blind, Placebo-Controlled Study of Tasquinimod in Men With Metastatic Castrate Resistant Prostate Cancer	Phase III	Tasquinimod versus placebo
NCT01188187	A Randomized Phase 3 Study Comparing Standard First-Line Docetaxel/Prednisone to Docetaxel/Prednisone in Combination With Custirsen (OGX-011) in Men With Metastatic Castrate Resistant Prostate Cancer	Phase III	Custirsen, docetaxel, prednisone versus docetaxel, prednisone
NCT02023697	A Three Arm Randomized, Open-label Phase II Study of Radium-223 Dichloride 50 kBq/kg (55 kBq/kg After Implementation of NIST Update) Versus 80 kBq/kg (88 kBq/kg After Implementation of NIST Update), and Versus 50 kBq/kg (55 kBq/kg After Implementation of NIST Update) in an Extended Dosing Schedule in Subjects With Castration-resistant Prostate Cancer Metastatic to the Bone	Phase II	Radium-223 dichloride standard versus high versus extended standard doses
NCT01212991	Prevail: A Multinational Phase 3, Randomized, Double-blind, Placebo-controlled Efficacy And Safety Study Of Oral Mdv3100 In Chemotherapy-naïve Patients With Progressive Metastatic Prostate Cancer Who Have Failed Androgen Deprivation Therapy	Phase III	Enzalutamide versus placebo
NCT01685983	A Phase 2 Open Label Study of Abiraterone Acetate (JNJ-212082) and Prednisolone in Patients With Advanced Prostate Cancer Who Have Failed Androgen Deprivation and Docetaxel-Based Chemotherapy	Phase II	Abiraterone versus prednisolone
NCT02003924	Prosper: A Multinational, Phase 3, Randomized, Double-blind, Placebo-controlled, Efficacy And Safety Study Of Enzalutamide In Patients With Nonmetastatic Castration-resistant Prostate Cancer	Phase III	Enzalutamide versus placebo
NCT01977651	A Multicenter, Single-Arm, Open-Label, Post-Marketing Safety Study to Evaluate the Risk of Seizure Among Subjects With Metastatic Castration-Resistant Prostate Cancer (mCRPC) Treated With Enzalutamide Who Are at Potential Increased Risk of Seizure	Phase IV	Enzalutamide
NCT02987543	A Phase III, Open Label, Randomized Study to Assess the Efficacy and Safety of Olaparib (Lynparza™) Versus Enzalutamide or Abiraterone Acetate in Men With Metastatic Castration-Resistant Prostate Cancer Who Have Failed Prior Treatment With a New Hormonal Agent and Have Homologous Recombination Repair Gene Mutations (PROfound)	Phase III	Olaparib versus enzalutamide or abiraterone acetate
NCT01188187	A Randomized Phase 3 Study Comparing Standard First-Line Docetaxel/Prednisone to Docetaxel/Prednisone in Combination With Custirsen (OGX-011) in Men With Metastatic Castrate Resistant Prostate Cancer	Phase III	Custirsen, docetaxel, prednisone versus docetaxel, prednisone
NCT02200614	A Multinational, Randomised, Double-blind, Placebo-controlled, Phase III Efficacy and Safety Study of BAY1841788 (ODM-201) in Men With High-risk Non-metastatic Castration-resistant Prostate Cancer	Phase III	BAY1841788 (ODM-201) versus placebo
NCT02257736	A Phase 3 Randomized, Placebo-controlled Double-blind Study of JNJ-56021927 in Combination With Abiraterone Acetate and Prednisone Versus Abiraterone Acetate and Prednisone in Subjects With Chemotherapy-naive Metastatic Castration-resistant Prostate Cancer (mCRPC)	Phase III	Apalutamide, abiraterone acetate, prednisone versus abiraterone acetate, prednisone
NCT00626548	A Phase III, Randomised, Placebo-controlled, Double-blind Study to Assess the Efficacy and Safety of Once-daily Orally Administered ZD4054 (Zibotentan) 10 mg in Non-metastatic Hormone-resistant Prostate Cancer Patients	Phase III	Zibotentan versus placebo
NCT00554229	A Phase III Trial to Test the Efficacy of ZD4054(Zibotentan), an Endothelin A Receptor Antagonist, Versus Placebo in Patients With Hormone Resistant Prostate Cancer (HRPC) and Bone Metastasis Who Are Pain Free and Mildly Symptomatic	Phase III	Zibotentan versus placebo
NCT02677896	A Multinational, Phase 3, Randomized, Double-blind, Placebo-controlled Efficacy and Safety Study of Enzalutamide Plus Androgen Deprivation Therapy (ADT) Versus Placebo Plus ADT in Patients With Metastatic Hormone Sensitive Prostate Cancer (mHSPC)	Phase III	Enzalutamide, androgen deprivation therapy versus placebo, androgen deprivation therapy
NCT01217697	An Open Label Study of Abiraterone Acetate in Subjects With Metastatic Castration-Resistant Prostate Cancer Who Have Progressed After Taxane-Based Chemotherapy	EAP	Abiraterone acetate versus prednisone

### Data collection and definitions

The patients’ clinical and pathological characteristics at CRPC diagnosis were retrieved from the institutional electronic medical record database. The obtained data included patient age; body mass index; serum PSA level at CRPC diagnosis; Gleason score; AJCC stage; previous local treatments received; Charlson Comorbidity Index (CCI); Eastern Cooperative Oncology Group performance score (ECOG PS); the site of metastasis; duration of docetaxel, abiraterone, enzalutamide, cabazitaxel, and radium-223 dichloride administration; docetaxel to androgen receptor axis-targeted agent sequencing; and laboratory values including peripheral blood hemoglobin, albumin, and alkaline phosphatase levels, and white blood cell counts.

CRPC was defined and evaluated according to the criteria of the Prostate Cancer Clinical Trials Working Group 2 [17]. The CSS interval was defined as the interval from the date of initial CRPC diagnosis to the date of death from prostate cancer. Patient survival and causes of death were investigated based on the National Cancer Registry Database or institutional electronic medical records.

### Statistical analysis

Clinicopathologic data were compared between CT participants and non-participants using descriptive statistics. Fisher’s exact test and the chi-squared test were used to compare categorical variables. The Mann-Whitney U-test was used to compare continuous variables across dichotomous categories. Kaplan-Meier curves were used to estimate CSS according to CT participation, with *p*-values computed using the log-rank test.

Univariate and multivariate Cox proportional hazards regression analyses were used to adjust for potential confounders in predicting CSS. All covariates with significant *p*-values in the univariate model were included in the multivariate model. Statistical analyses were performed using IBM SPSS software (version 23; IBM Corp., Armonk, NY, USA). All tests were two-tailed, with statistical significance set at a *p*-value of < 0.05.

## Results

### Baseline characteristics

The baseline clinical and pathological features of the overall population and of the subgroups stratified by CT participation are presented in Table 2. Of the 299 patients, 65 (21.7%) participated in CTs pertaining to systemic treatment target at CRPC while 234 (78.3%) received pre-established, standard systemic treatment outside of a CT setting. A lower proportion of CT participants had ECOG PS ≥2 at CRPC diagnosis than did non-participants (4.6% vs. 14.9%; *p* = 0.033), while PSA levels at CRPC diagnosis were lower in CT participants compared to non-participants (25.8 ng/mL vs. 88.4 ng/mL; *p* = 0.005). Distributions of potential survival prognosticators of CRPC, namely, age, body mass index, TNM stages, Gleason score, metastatic sites, and CCI were comparable between the two groups.

**Table 2:** Clinicopathologic characteristics of castration-resistant prostate cancer patients, stratified by clinical trial participation

	Overall (n = 299)	Clinical trial		
		Participants (n = 65)	Non-participants (n = 234)	*p*
Age	66.5 (61.0–71.8)	65.0 (62.0–71.0)	67.0 (61.0–72.0)	0.384
Body mass index	23.1 (20.9–24.7)	22.9 (21.3–24.7)	23.4 (21.2–25.1)	0.345
Laboratory values^a^				
PSA (ng/mL)	69.2 (15.0–182.0)	25.8 (9.6–73.6)	88.4 (18.0–247.3)	0.005
Hemoglobin (g/dL)	12.0 (10.7–13.0)	12.4 (11.7–13.3)	11.9 (10.4–12.9)	0.514
Albumin (U/L)	4.0 (3.7–4.4)	4.3 (4.0–4.5)	4.0 (3.7–4.3)	0.001
ALP (U/L)	109.0 (70.0–209.0)	88.0 (67.0–133.5)	118 (71.0–221.5)	0.070
WBC count (× 10^9^/L)	5.8 (4.8–7.3)	5.8 (4.7–7.3)	5.8 (4.9–7.3)	0.919
T stage				0.764
≤ T2	187 (62.5%)	42 (64.7%)	145 (61.9%)	
≥ T3	112 (37.5%)	23 (35.3%)	89 (38.1%)	
N stage				0.491
N0	127 (42.5%)	30 (46.2%)	97 (41.5%)	
N1	172 (57.5%)	35 (53.8%)	137 (58.5%)	
M stage				1.000
M0	73 (24.4%)	15 (23.1%)	58 (24.8%)	
M1	226 (75.6%)	50 (76.9%)	176 (75.2%)	
Metastatic site				
Bone	166 (55.5%)	37 (57.0%)	129 (55.1%)	0.856
Visceral	8 (2.7%)	8 (12.3%)	0 (0.0%)	0.215
Lymph node	142 (47.5%)	26 (40.0%)	116 (49.6%)	0.116
Gleason score				0.267
≤ 7	156 (52.2%)	30 (46.2%)	126 (53.8%)	
≥ 8	143 (47.8%)	35 (53.8%)	108 (46.2%)	
CCI				0.780
≤ 1	142 (%)	32 (49.2%)	110 (47.0%)	
≥ 2	157 (%)	33 (50.8%)	124 (53.0%)	
ECOG PS				0.033
≤ 1	261 (87.3%)	62 (95.4%)	199 (85.1%)	
≥ 2	38 (12.7%)	3 (4.6%)	35 (14.9%)	
Primary treatment^b^				
Prostatectomy	149 (49.8%)	47 (72.3%)	102 (43.6%)	< 0.001
Radiation therapy	33 (11.0%)	10 (15.4%)	23 (9.8%)	0.261

Data are presented as the median (interquartile range) or number (%)

^a^At diagnosis of castration-resistant prostate cancer

^b^Number of primary treatment does not sum to 299 patients due to the existence of men who did not receive any local treatment with curative intent

Abbreviations: *ALP* alkaline phosphatase, *CCI* Charlson Comorbidity Index, *ECOG PS* Eastern Cooperative Oncology Group performance status, *PSA* prostate-specific antigen, *WBC* white blood cell

The treatments administered for CRPC are described in Table 3. CT participants received significantly more cycles of docetaxel than did non-participants managed outside the CT setting. Because of Korea’s National Health Insurance policy of providing reimbursement for enzalutamide used for post-chemotherapy patients with CRPC, enzalutamide was predominantly used in the post-docetaxel setting. There were no differences between the two groups in terms of the proportions of other systemic treatments used.

**Table 3:** Treatments administered for castration-resistant prostate cancer

	Overall (n = 299)	Clinical trial		
		Participants (n = 65)	Non-participants (n = 234)	*p*
Docetaxel				
N	242 (80.9%)	41 (63.1%)	201 (85.9%)	0.001
No. cycles	4.0 (2.0–9.0)	7.0 (4.0–12.5)	4.0 (2.0–9.0)	0.003
ARAT agent use				
Pre-chemotherapy				0.502
Abiraterone	10 (3.3%)	2 (3.1%)	8 (3.4%)	
Enzalutamide	15 (5.0%)	4 (6.2%)	11 (4.7%)	
Post-chemotherapy				< 0.001
Abiraterone	23 (7.7%)	13 (20.0%)	10 (4.3%)	
Enzalutamide	108 (36.1%)	9 (13.8%)	99 (42.3%)	
Cabazitaxel	1 (0.3%)	0 (0.0%)	1 (0.4%)	1.000
Radium-223	5 (1.7%)	3 (4.6%)	2 (0.9%)	0.070

Abbreviations: *ARAT* androgen receptor axis-targeted

### Survival outcome according to clinical trial participation

**Fig. 1:**
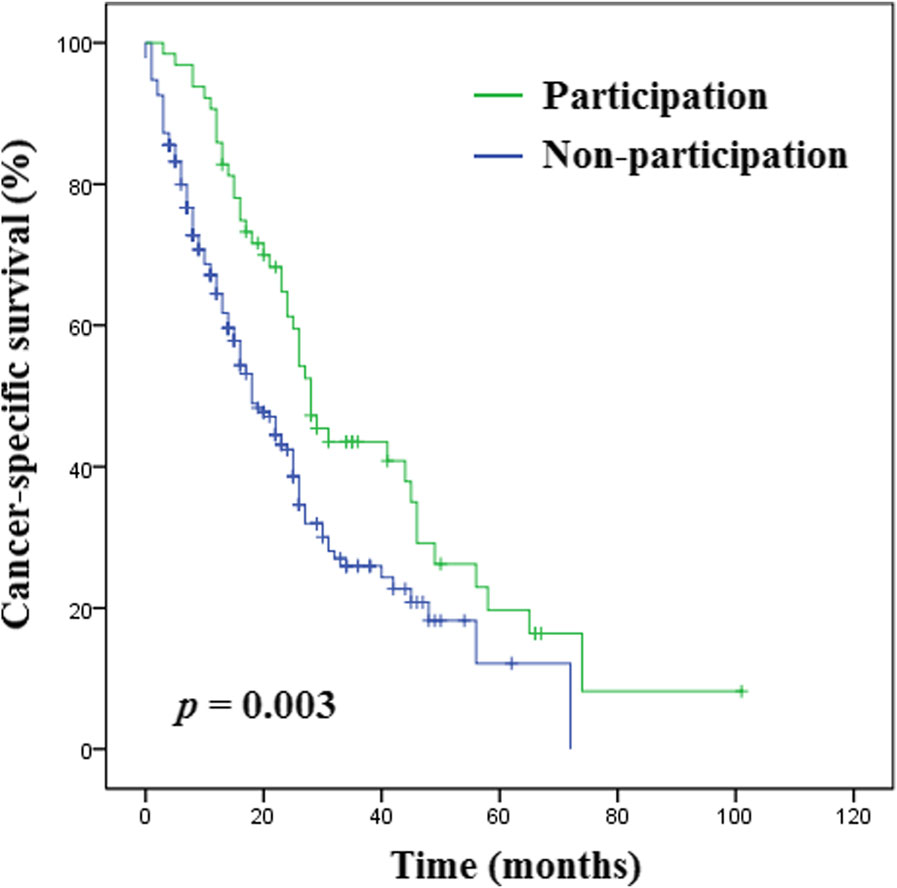
Cancer-specific survival of patients with castration-resistant prostate cancer, stratified by clinical trial participation versus non-participation

Survival results as of September 2017 were used in this analysis and are presented in Table 4 and Fig. 1. During the median follow-up period of 16.0 months, the median CSS interval was 13.0 months. Overall, 187 (66.3%) cancer-specific deaths were noted, which translated to a 2-year CSS rate of 46.8%. CT participants exhibited significantly higher 2-year CSS rates than non-participants (61.3% vs. 42.4%; *p* = 0.003).

**Table 4:** Survival outcomes of patients with castration-resistant prostate cancer, stratified by clinical trial participation

	Overall (n = 299)	Clinical trial		
		Participants (n = 65)	Non-participants (n = 234)	*p*
No. cancer-specific deaths	187 (62.5%)	44 (67.7%)	143 (61.1%)	0.364
2-year cancer-specific survival	46.8%	61.3%	42.4%	0.003
CRPC to death (months)	13.0 (6.0–24.3)	23.5 (13.3–30.5)	11.0 (5.0–19.3)	< 0.001
Total follow-up (months)	16.0 (7.2–26.0)	26.0 (16.0–39.8)	13.5 (6.0–24.0)	< 0.001

Data are presented as number (%) or median (interquartile range)

Abbreviations: *CRPC* castration-resistant prostate cancer

### Predictors of cancer-specific survival

Predictors of CSS are presented in Table 5. Univariate Cox-regression analyses demonstrated that patient age, PSA level at CRPC diagnosis, albumin and alkaline phosphatase levels, biopsy Gleason score ≥ 8, ECOG PS ≥2, less number of docetaxel cycles administered, and non-participation in CTs were associated with lower risk of CSS. Multivariate analysis revealed that PSA at CRPC diagnosis, alkaline phosphatase level, biopsy Gleason score ≥ 8, ECOG PS ≥2, less number of docetaxel cycles administered, and non-participation in CTs independently predicted lower risk of CSS.

**Table 5:** Predictors of cancer-specific mortality in patients with castration-resistant prostate cancer

	Univariate			Multivariate		
	HR	(95% CI)	*P*	HR	(95% CI)	*p*
Age	1.038	(1.016–1.061)	0.001	1.020	(0.998–1.042)	0.069
Body mass index	0.968	(0.901–1.041)	0.382			
PSA^a^	1.001	(1.000-1.001)	< 0.001	1.001	(1.000–1.001)	0.018
Hemoglobin^a^	1.001	(0.999-1.002)	0.306			
Albumin^a^	0.408	(0.301-0.553)	< 0.001			
Alkaline phosphatase^a^	1.001	(1.000–1.001)	< 0.001	1.001	(1.001–1.002)	< 0.001
T stage (≥T3 vs. ≤T2)	0.865	(0.524–1.430)	0.271			
N stage (1 vs. 0)	1.251	(0.922–1.697)	0.152			
M stage (1 vs. 0)	1.528	(0.983–2.376)	0.062			
Gleason score (≥8 vs. ≤7)	1.957	(1.441–2.658)	< 0.001	2.004	(1.452–2.767)	< 0.001
CCI ≥4	1.197	(0.811–1.765)	0.365			
ECOG ≥2	1.802	(1.216–2.670)	0.003	1.304	(1.164–2.158)	0.035
Docetaxel cycles	0.926	(0.900–0.953)	0.026	0.943	(0.915–0.972)	0.011
Primary treatment						
Prostatectomy	1	(reference)				
Radiation therapy	0.778	(0.580–1.141)	0.584			
ARAT agent sequencing						
Pre-chemotherapy	1	(reference)				
Post-chemotherapy	0.865	(0.524–1.430)	0.572			
Radium-223 administration	0.803	(0.255–2.527)	0.707			
Clinical trial participation	0.593	(0.417–0.843)	0.004	0.585	(0.429–0.797)	0.038

^a^Laboratory values at diagnosis of castration-resistant prostate cancer

Abbreviations: *ARAT* androgen receptor axis-targeted, *CI* confidence interval, *CCI* Charlson Comorbidity Index, *ECOG PS* Eastern Cooperative Oncology Group performance status, *HR* hazard ratio, *PSA* prostate-specific antigen

## Discussion

Systemic treatment for CRPC has rapidly evolved. Identifying patients for judicious application of optimal treatment strategies is imperative in the current era of multidisciplinary treatment options. However, the selection of agents is often limited by the availability of novel agents and reimbursement issues. In this regard, participation in CTs may provide a breakthrough opportunity for access to innovative therapeutic approaches in addition to third party payer coverage. Participation in CTs is based on the patient’s notion that a survival benefit can be achieved. Our study demonstrated that participation in CTs pertaining to CRPC agents, compared with non-participation, may improve CSS regardless of metastatic status.

The biological mechanisms underlying improved CSS observed with CT participation in our study is unclear; however, several reasons have been proposed. First, an experimental treatment effect may have existed, in which CT participants received better treatment in early-phase CTs than they would have received with standard therapies [9]. This effect may potentially have affected our results in that systemic agents that have been identified to prolong survival—namely, abiraterone, enzalutamide, docetaxel, and radium-223 dichloride—were included in either the experimental or control arms in 66% of CTs included in our analysis. Furthermore, potential selection bias may arise from the 32 patients who were excluded from final analysis, if these patients had been allocated to receive novel agents without proven survival benefit. However, among the four excluded patients in the CT participation group, three patients had received abiraterone or docetaxel while one patient was blinded to arm allocation, precluding any alteration in our study results.

Second, a participation effect may have existed, in which aspects of CT participation other than exposure to investigational therapy may have improved outcomes [14]. Specifically, the participation effect comprises the following: 1) a protocol effect regarding the way the treatments are delivered; 2) a care effect including incidental aspects of care; 3) the Hawthorne effect, which is initiated by changes in physician or patient behavior in regard to the perception that they are under observation; and 4) the placebo effect, which mediates the psychological behavior of the participant based on the awareness that they are beneficiaries of therapeutic advances [14, 16].

Third, the improved survival outcomes observed with CT participation in our study may have resulted from inherent differences in baseline patient and tumor features. In our study, the performance status, as well as PSA and albumin levels of patients who participated in CTs were more favorable than those of non-participants; this might have affected the results. However, the proportions of potential survival confounders including age, tumor stage and grade, metastatic burden, and comorbidities were comparable between the groups. To overcome the challenge in separating possible true effects from false effects of the discrepancy in baseline patient and tumor features, statistical adjustments were made for a comprehensive set of confounders of survival among patients with CRPC, to confirm that CT participation itself was an independent prognosticator. Another selection bias arises from the effect of protocol eligibility criteria [12]. Most CTs included in our study strictly prohibited enrollment of patients with advanced disease, such as those with brain metastasis, an adverse prognostic factor for several cancers [18–20]. However, the two study groups had comparable proportions of metastatic location and burden, corroborating our hypothesis.

Fourth, a bias in data collection with regard to survival may have affected outcomes; survival follow-up could be more completely censored in CT participants than in non-participants. Moreover, patients in the advanced stages of the disease who participated in CTs could have been inherently more adherent to treatment follow-up schedules, whereas non-participants might opted for supportive care even if anti-cancer treatment may have prolonged survival [16, 21].

The present study revealed that CT participants received more docetaxel cycles than non- participants. Docetaxel remains the standard treatment for metastatic CRPC and has been the mainstay for CTs of sequential strategies since its approval in 2004 [22]. The improved survival in CT participants may be attributed to better chemotherapy efficacy and subsequent prolonged duration of docetaxel administration, as shown in multivariate analysis. Our study also demonstrated that Gleason score, and PSA and alkaline phosphatase levels at CRPC diagnosis are independent predictors of CSS. These findings compare favorably to those of previous retrospective studies that investigated prognosticators for survival in patients with metastatic CRPC, which implies that our cohort ably represented the whole population of the disease status and that our conclusions are generalizable [23, 24].

With our use of retrospective data, it is difficult to determine which of the abovementioned effects contributed to the survival benefit associated with CT participation. Indeed, a randomized controlled trial in which patients are randomized to be offered CT participation would be warranted to ensure baseline comparability and to investigate potential confounders. However, if at least one of the abovementioned effects may have truly affected improved CSS outcome in our CT participants, it would provide evidence to offer CT participation whenever indicated to patients with CRPC for its inherent survival advantage.

The strengths of the current study include the incorporation of comprehensive survival prognosticators of CRPC, including patient and tumor characteristics, comorbidities, performance status, laboratory values, and treatment information that were available for all patients. Furthermore, CT participants included in our study received novel hormonal, chemotherapeutic, and radiopharmaceutical therapeutic agents approved in the last 8 years, which suggests that our results are applicable in this contemporary era of multidisciplinary treatment strategies. At the same time, several limitations are worth mentioning. First, selection bias may have existed due to the retrospective nature of the study. This study was a non-randomized study; therefore, there was a lack of a standard therapeutic approach in which physician and patient preferences existed regarding the implementation of a CT. Moreover, a discrepancy existed in treatment protocols used in various CTs, such as the frequency of imaging and laboratory testing, and between each physician who treated patients with standard care. Second, the existence of unaccounted imbalances in baseline patient and tumor characteristics cannot be overlooked. However, these potential baseline discrepancies which may have affected our outcomes were accounted for, and our results were derived from multivariate Cox-regression analyses. Lastly, we did not account for the data of patients who participated in CTs but later declined to continue and opted for best supportive care, which may have affected survival outcomes. The abstract of this article was presented at the 33rd Annual EAU Congress [25].

## Conclusions

This observational study provides novel findings that the CSS outcomes of patients diagnosed with CRPC who participated in CTs were better than those of non-participants who received pre-established, standard systemic therapy outside of a CT setting. Our findings imply that CT participation is associated with CSS, and that CT participation should be offered to patients with CRPC whenever indicated.

## Abbreviations

CCI: Charlson Comorbidity Index
CRPC: Castration-resistant prostate cancer
CSS: Cancer-specific survival
CT: Clinical trial
ECOG PS: Eastern Cooperative Oncology Group performance score
PSA: Prostate-specific antigen
